# Evaluating Student Communication Skills in a Simulation Setting: A Trends Analysis

**DOI:** 10.1002/jdd.70042

**Published:** 2025-09-26

**Authors:** Tamanna Tiwari, Holly Nowak, Tanya Russell, Austin Albino, William Rivera, Tamara Tobey, Bruce A. Dye

**Affiliations:** ^1^ Department of Primary Dental Care, School of Dentistry University of Minnesota Minneapolis USA; ^2^ School of Dental Medicine University of Colorado Anschutz Medical Campus Aurora Colorado USA; ^3^ Center for Advancing Professional Excellence (CAPE) University of Colorado Anschutz Medical Campus Aurora Colorado USA; ^4^ Colorado Public Health School, University of Colorado Anschutz Medical Campus Aurora Colorado USA

**Keywords:** clinical competence, patient simulation, standardized patients

## Abstract

**Objective:**

This study aims to comprehensively evaluate the communication skills of dental students at the University of Colorado over a 7‐year period (2016–2022). The study hypothesis is that communication skills will remain consistent over the 7‐year period. This study uses standardized patients (SPs) from the Center for Advanced Professional Excellence (CAPE) and the Interprofessional Anschutz Communication Skills Toolbox (I‐ACT) to assess students' communication skills.

**Methods:**

A longitudinal analysis of third‐year dental students and first‐year advanced‐standing students interacting with SPs was conducted. SPs utilized the I‐ACT toolbox to assess students' communication skills. Fifteen questions were designed to evaluate key aspects of communication. SPs scored student performance using a scale of 1.0 (*Yes*), 0.5 (*Partial*), and 0.0 (*No*). Data were collected from 2016 to 2022 and analyzed using descriptive statistics and linear regression, with time as the dependent variable.

**Results:**

The analysis showed consistent performance in the “Introduction,” “Sharing Information,” and “Sustaining Relationship” domains, with low *F*‐values indicating minimal variance over time. However, the “Gathering Information,” “Sustaining Structure,” and “Closing Session” domains exhibited notable variability. The “Closing Session” domain, particularly for Patient 3, showed the highest variability with substantial changes in student performance over time in a positive direction.

**Conclusion:**

The study highlights strengths and areas for improvement in dental students' communication skills. Consistent performance in several communication domains suggests that current training methods are effective.

## 1 Introduction

Clinical simulations, a technique for replicating real‐world healthcare scenarios, are widely used in health professional education to teach and assess students’ cognitive, psychomotor, and affective skills [1]. These simulation activities give students the unique opportunity to practice specialized scenarios, such as managing life‐threatening and emergency situations [2], handling surgical complications [3], practicing in unfamiliar environments [4, 5], and promoting cultural sensitivity [6] in a safe and controlled space. Standardized patients (SPs) are one of the many methods used to teach and assess skills in clinical simulations. An SP is an actor trained to portray all aspects of an actual patient and interact with students in a case scenario [7]. SPs create realistic provider‐patient scenarios by allowing students to interact with the entirety of actual patients, including their stories, physical symptoms, emotional responses to illness, attitudes toward doctors, and stress coping with disease [8]. SP methodology enables students to practice real‐world healthcare scenarios and provides them with the opportunity to receive patient‐centered feedback from SPs and instructors, further enhancing their learning [9, 10].

Medical schools and the military are institutions that have historically utilized various simulations for medical training purposes [11, 12]. The use of this training methodology can be traced back to 1963 when Howard S. Barrows used “programmed patients” to teach third‐year neurology clerks [13]. Research shows that SP methodology has been utilized across various healthcare professions and is particularly effective in teaching communication and interpersonal skills [2, 13, 14]. Current research demonstrates that incorporating SPs into medical schools enhances students’ communication and interpersonal skills, confidence, and clinical competence [15].

SP methodology in dental education is evolving. Some dental schools have incorporated SP methodology to train and evaluate students' skills in teamwork [9], conflict resolution [15], tobacco cessation counseling [16], and communication [11]. Evaluation of these skills through the use of SPs in objective structured clinical exams (OSCEs) is on the rise, as studies show this methodology helps students develop skills in decision‐making [17], communication [17], and self‐reflection [18]. Learning these skills is of paramount importance as healthcare providers with strong communication skills tend to have higher patient satisfaction [19], better patient‐health outcomes [20], reduced physician burnout [20], and better patient adherence [20]. Providers' communication and interpersonal skills, such as active learning, nonverbal communication, and empathy, are vital for understanding patient needs, building trust, and shared decision‐making in dental treatment [21, 22].

The University of Colorado School of Dental Medicine (CUSDM) has utilized a variety of SP methodologies for nearly a decade in partnership with the Center for Advancing Professional Excellence (CAPE), which is a full‐service education and assessment center at the University of Colorado Anschutz Medical Campus. The CAPE utilizes SPs to train and assess learners and practitioners in communication and other pertinent skills. In 2015, CAPE established the Interprofessional Anschutz Communication Toolbox (I‐ACT) to facilitate interprofessional collaboration in creating and refining communication skill descriptions for healthcare assessment, promoting a more precise understanding, modeling best practices, and advocating for a comprehensive, integrated communication curriculum [23].

While assessing activities and receiving feedback from actual or simulated dental patients can improve dental learners' communication, using simulated clinical environments supports learning in complementary domains to the clinician expert, significantly enhancing the humanistic elements over the more observable checklist content [24]. SP methodology is essential in teaching dental students these skills [22]. Although the utilization of SP methodology in dental education has been studied, there is a paucity of research evaluating dental student performance in clinical simulation using SPs over time. Our aim is to present an assessment of 7‐year trends in the communication skills of third‐year dental students and first‐year advanced‐standing students with SPs at the University of Colorado School of Dental Medicine.

## 2 Methods

This study received exempt status from the Colorado Multiple Institutional Review Board (COMIRB study number 19‐2158). CAPE is an educational resource that uses SPs to teach and evaluate the skills of health professional students and residents. CAPE established I‐ACT to enable interprofessional collaboration in creating and refining communication skill descriptions for healthcare assessment [23]. Program faculty from across disciplines were brought together to form a community of practice in assessing students' communication skills.

This study evaluated the performance trends of student interactions with three SP cases, referred to as Patients 1, 2, and 3, from 2016 to 2022. Case descriptions will follow later in Section 2. The central hypothesis of this study was that students' communication skills during interactions with standardized patients would remain consistent within each domain over a 7‐year period.

### 2.1 Description of the Activity

This SP activity served as a summative assessment for third‐year dental students and first‐year advanced‐standing students. Students practiced encounters with patients from diverse backgrounds and experiences, and were assessed on communication skills. In doing so, students strengthen these skills and help foster cultural competencies within their curricula. Each SP encounter lasted 15 min; after that, SPs scored the students using the I‐ACT Toolbox, assigning a score of 1.0 (*Yes*), 0.5 (*Partial*), or 0.0 (*No*) to all 15 questions. Students also received 7 min of reflective verbal feedback from the student performance specialists (SPs). Then, the students debriefed with a CAPE coach, who listened to their activity summaries and provided feedback on communication skills.

### 2.2 Questionnaire

The I‐ACT questions (Table 1) assessed the students' communication skills with patients, including greeting them and explaining the appointment agenda. The questionnaire assessed the students on exploring and identifying the patient's perspectives, tailoring information to their needs, balancing time and turn‐taking, and using names when addressing others throughout the conversation. Further, the questions evaluated the students' nonverbal behavior toward their patients, including eye contact, facial expressions, posture and position, and respecting and adapting to other person's circumstances. The questionnaire concluded with the students' approach to summarizing the following steps, closing interactions, and requesting any other questions. Lastly, a global items category was included, enabling subjective and objective comments on listening to patient requests and perspectives, as well as tailoring actions and care plans to meet mutual needs and capacities.

**TABLE 1:** Questions utilized in the I‐ACT and corresponding categories.

**Questions**	**Domains**	**Domain details**
Greets Appropriately: Introduces self and role and confirms patient name.	Domain 1	Introduction/initiating interaction
Sets Mutual Agenda: Seeks the other person's understanding of the situation to clearly identify and confirm the reason for interaction.	Domain 1	Introduction/initiating interaction
Clarifies and Balances Narrative Details: Appropriately uses closed‐ended questions to clarify details of the story (e.g., dates, sequence, onset, location, and details) and balance interaction.	Domain 2	Gathering information
Explores Other Person's Perspective: Fully elicits other people's ideas, concerns, expectations, beliefs, and dimensions of life context to understand perspective and position.	Domain 2	Gathering information
Identifies and Tailors Information to Other Person's Needs: Asks what the other person knows, has seen/read/ heard, and adapts information through the appropriate use of language (or models and diagrams) to meet their needs.	Domain 3	Sharing information
Practices Chunks and Checks for Understanding: Consistently shares information tailored to the other person's needs and capacities and checks for understanding (ask questions, express doubts).	Domain 3	Sharing information
Balancing Talk Time/Turn Talking: Use reflective listening, summaries, and/or signposts appropriately to avoid verbal dominance and distraction by any person in the interaction.	Domain 4	Sustaining structure
Attends to Timing: Structures interaction appropriately to complete all tasks and topics for inquiry in the time allotted.	Domain 4	Sustaining structure
Appropriate Nonverbals: Consistently demonstrates appropriate nonverbal behavior (incl. eye contact, facial expression, posture, position and movement, vocal rate, volume, tone, use of notes, and computers without interference).	Domain 5	Sustaining relationship
Respects and Adapts to Others Person's Circumstances: Verbally and nonverbally attends to other person's circumstances (physical/mental/social/emotional) and adapts communication style/approach as appropriate.	Domain 5	Sustaining relationship
Demonstrates Empathy: Recognizes and acknowledges emotional state of self, others, situation expressing care, understanding of the patient's experience and concern verbally and nonverbally.	Domain 5	Sustaining relationship
Summarizes Discussions and Next Steps: Provides a brief summary of interaction and reviews immediate and future steps.	Domain 6	Closing the session
Final Check: Closes interactions with a final check on an understanding of the plan and request for any other questions.	Domain 6	Closing the session
Global Item: This person sought and listened to my requests and perspective, tailoring our action/care plan to meet our mutual needs and capacities.	Domain 6	Global/overall

### 2.3 Standardized Patients (SPs) Cases

Patient 1 was a 70‐year‐old man with a history of left‐hemispheric stroke who presented for examination and cleaning. The stroke resulted in significant right‐sided weakness affecting his face and upper and lower extremities, as well as expressive aphasia. The patient needed to use a wheelchair, and when transferring from the dental chair to the wheelchair was “guarded” by the dental provider. Although the patient had occasional difficulties with word‐finding and clearly articulating some words, he was communicative and coherent in his speech. The patient reported that prior to his stroke, a year before, he was meticulous about his oral hygiene but has since struggled with routine oral hygiene as he did not receive regular support with toothbrushing. This created concerns for bad breath, periodontal disease, and decay to/around his crowns and veneers. In addition to his routine exam, the patient hoped for guidance in managing his oral hygiene after limited instruction/assistance from his physical therapist. The patient was a retired law professor with dental insurance.

Patient 2 was a 30‐ to 50‐year‐old pregnant woman at 18 weeks of gestation who presented with concerns about a broken right lower molar filling and a dental history notable for extensive caries and fillings. The patient reported she immigrated to the United States as a teenager and came from a culture with a belief that pregnant mothers should not receive dental care. She described a substantial value for family recommendations and advice. Given this background, she preferred to delay nonessential care. She wished to address the sharp edge of the filling and explore protective options to avoid further damage to her tooth. While the patient complained of mild pain and sensitivity with exposure to cold food or beverages, her primary worry was the health and safety of her child.

Patient 3 is a 45‐ to 50‐year‐old man who presents to the office complaining of sudden onset, severe, sharp, right lower back tooth pain after eating corn nuts. Aside from sore gums and occasional gum bleeding with toothbrushing, he denies any other oral health concerns. The patient has a history of hypertension, for which he has elected not to continue treatment with antihypertensive medications due to their side effects. He is hypertensive on arrival with a significantly elevated BP of 174/100. In addition to his history of hypertension, the patient reports a history of elevated blood glucose levels and symptoms of increased urinary frequency and polydipsia. His family history is notable for hypertension, coronary artery disease, and diabetes. The patient is from a low‐income background and does not have dental insurance.

### 2.4 Data Analysis

The data was collected by SPs as part of the student evaluation from 2016 to 2022 and imported into an Excel file. Data management and analyses were performed in SAS v9.4 (SAS Institute, Cary, NC). Descriptive statistics were run for each of the cases for each year. Linear regression was run over time to test the differences in student performance in all three cases. Each of the six performance categories was used as an independent variable, and time (2016–2022) was the dependent variable. The data was visualized via three graphs based on the regression for each case.

## 3 Results

Each student completed two SP activities. Patient 1 was used for the student–SP communication evaluation from 2016 to 2019, Patient 2 from 2016 to 2022, and Patient 3 from 2020 to 2022. Table 2 presents the descriptive analysis of all three patients over the years, along with the average scores for each year and each of the skills assessment questions, categorized into six performance domains. A total of 827 students were evaluated over the course of these 7 years. All 827 students participated in the Patient 2 activity, 451 for Patient 1 and 357 for Patient 3.

**TABLE 2:** Descriptive data for each patient under all categories by year*.

	Patient 1 (*N* = 451)				Patient 2 (*N* = 827)							Patient 3 (*N* = 357)		
Mean values for categories	2016	2017	2018	2019	2016	2017	2018	2019	2020	2021	2022	2020	2021	2022
Introduction	0.90	0.94	0.91	0.92	0.90	0.88	0.91	0.91	0.92	0.96	0.97	0.85	0.95	0.93
Gathering information	0.87	0.92	0.81	0.86	0.87	0.71	0.78	0.82	0.82	0.88	0.92	0.89	0.88	0.90
Sharing information	0.79	0.86	0.78	0.81	0.79	0.72	0.77	0.78	0.78	0.84	0.92	0.84	0.83	0.84
Sustaining structure	0.86	0.95	0.80	0.84	0.86	0.61	0.71	0.73	0.83	0.86	0.85	0.86	0.89	0.88
Sustaining relationship	0.89	0.89	0.81	0.87	0.89	0.81	0.79	0.80	0.97	0.96	0.98	0.93	0.96	0.95
Closing session	0.70	0.80	0.87	0.87	0.70	0.77	0.78	0.81	0.91	0.92	0.94	0.77	0.95	0.86
Global	0.91	0.96	0.92	0.91	0.91	0.83	0.87	0.88	0.97	0.95	0.99	0.95	0.97	0.95

* Provides an overview of dental students' performance across 15 skills assessment questions, categorized into six performance domains. Data are presented for each year the case study was conducted, with students being evaluated on their demonstration of skill mastery. Scores were assigned on a scale of 0–1, where a score of 1.0 indicated “*Yes*,” 0.5 indicated “*Partial*,” and 0.0 indicated “*No*.”

**FIGURE 1:**
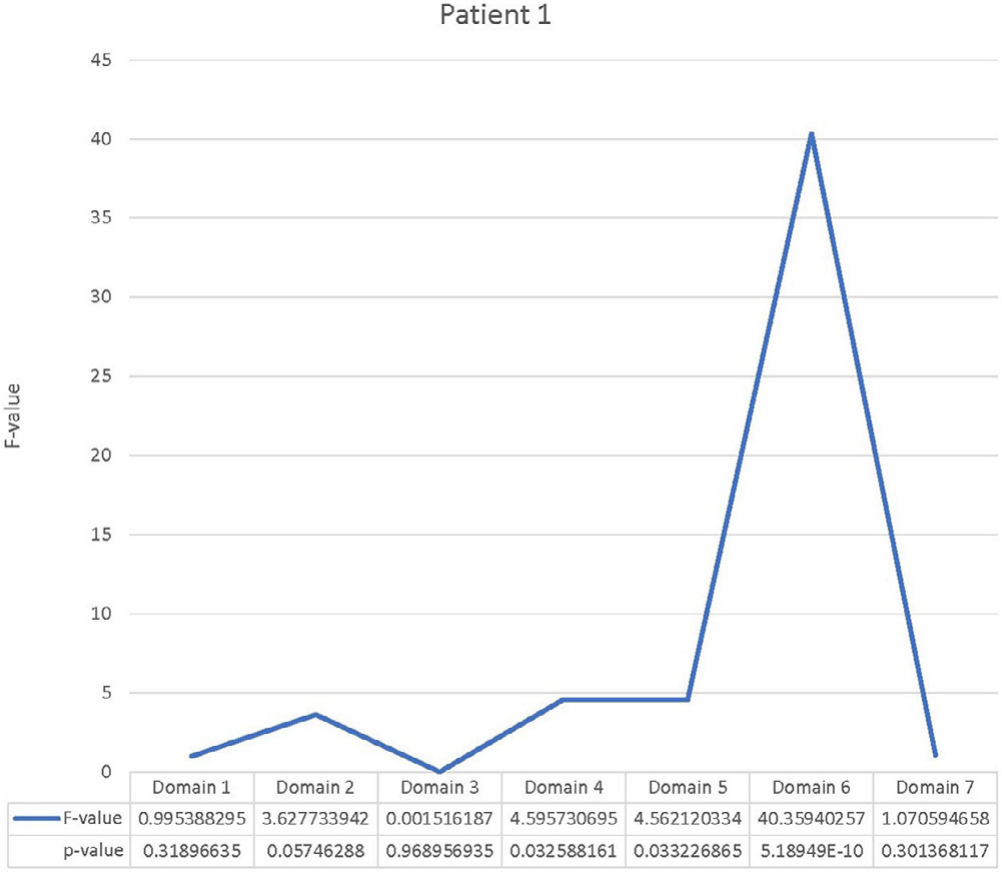
Evaluation of student and SP communication results for Patient 1 over time (measured by linear regression). Domain 1 = introduction/initiating interaction; Domain 2 = gathering information; Domain 3 = sharing information; Domain 4 = sustaining structure; Domain 5 = sustaining relationship; Domain 6 = closing session; Domain 7 = global/overall.

**FIGURE 2:**
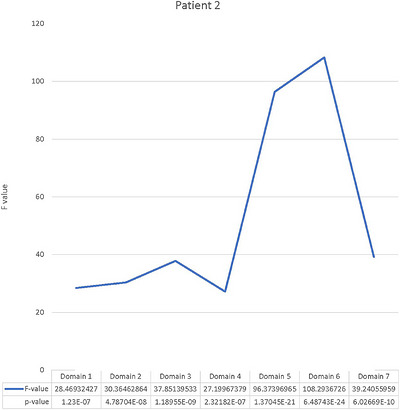
Evaluation of student and SP communication results for Patient 2 over time (measured by linear regression). Domain 1 = introduction/initiating interaction; Domain 2 = gathering information; Domain 3 = sharing information; Domain 4 = sustaining structure; Domain 5 = sustaining relationship; Domain 6 = closing session; Domain 7 = global/overall.

**FIGURE 3:**
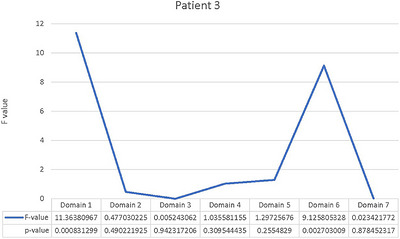
Evaluation of student and SP communication results for Patient 3 over time (measured by linear regression). Domain 1 = introduction/initiating interaction; Domain 2 = gathering information; Domain 3 = sharing information; Domain 4 = sustaining structure; Domain 5 = sustaining relationship; Domain 6 = closing session; Domain 7 = global/overall.

Figures 1, 2, 3 present the assessment data for student–SP communication in each patient scenario, using regression analysis over time. Each performance domain represents a critical aspect of communication, and the analysis highlights significant trends over the specified period. These results highlight strengths and areas for improvement in student–SP communication over the years.

Figure 1 shows the regression analysis results for student–SP communication evaluation for Patient 1. The significantly high consistency in the sustaining structure (*F* = 4.59; *p* = 0.033) and sustaining relationship (*F* = 4.56; *p* = 0.033) domains reflected a strong student–SP interaction over the years. The closing session domain also showed a significant model (*F* = 40.36; *p* = 5.18949E‐10), indicating a good model fit. However, the significant variability over the years in the closing session domain suggests that students have shown higher variability in communication within this category over time, as the scores have increased; this is a positive trend. This further means that students' performance in this area has steadily improved, indicating a positive trajectory in learning outcomes.

Figure 2 shows the regression analysis results for student–SP communication evaluation for Patient 2. The introduction (*F* = 28.467; *p* = 1.23E‐07), gathering information (*F* = 30.36; *p* = 4.78E‐08), and sharing information (*F* = 37.85; *p* = 1.18E‐09) domains were significant and relatively low, indicating consistency over time. This demonstrates a consistent approach to initiating patient interactions with Patient 2 over the years, indicating that students have developed a stable and consistent method for initiating patient interactions in this case. In addition, this implies that the training for early‐stage communication (introductions, history taking, and explaining information) is effective and standardized across different student cohorts. The sustaining structure domain showed a significant moderate *F*‐value (*F* = 27.19; *p* = 2.32E‐07), reflecting some variability in maintaining the session's structure over the years. The sustaining relationship (*F* = 96.37; *p* = 1.37E‐21) and closing session (*F* = 108.29; *p* = 6.49E‐24) domains exhibited the highest *F*‐value among all domains, indicating substantial variability in the positive direction, suggesting that students are making considerable strides in rapport building and effective session closure. While the variability is statistically significant, it reflects positive growth, with students showing improved emotional engagement and professionalism over time.

Figure 3 shows the regression analysis results for student–SP communication evaluation for Patient 3. The introduction domains showed high consistency in initiating patient interactions over time (*F* = 11.36; *p* < 0.0008). The closing session domain was also statistically significant (*F* = 9.13; *p* < 0.003) and exhibited some variability in a positive direction, indicating improvement over time and suggesting growing competency in concluding patient interactions in a professional and empathetic manner.

## 4 Discussion

The results of this study offer significant insights into the performance trends of dental students' communication skills with SPs from diverse backgrounds over a 7‐year period. The central hypothesis of this study was that performance would remain consistent over time. The findings reveal a more nuanced pattern: some domains indeed demonstrated consistency, while others exhibited statistically significant variability that reflects growth in a positive direction. The observed year‐over‐year improvement in certain domains suggests that students are continuing to develop their interpersonal and clinical communication skills as they progress through the curriculum. These are complex skills that naturally require more practice and refinement, especially as students gain confidence in the person‐centered care model. These observed trends could stem from variations in training emphasis on communication across different cohorts, individual differences in students' comfort levels, and skills in concluding patient encounters. The growth was observed in the closing of a session, which effectively requires more nuanced skills (e.g., summarizing, confirming understanding, and ending on a reassuring note), which students develop over time [24, 25].

This simulation activity also provides insights into the students' performance as evaluated by the SPs—a critical and transparent methodological approach to grading. Past research has shown that students are more likely to accept feedback from SPs than from faculty. Thus, SP methodology is an established learning approach in communication skills for healthcare students, and it can foster a practical learning environment for teaching person‐centered care [26, 27].

These results also relate to the strengths and areas for improvement in teaching communication skills in the curriculum and student training methodologies at the University of Colorado School of Dental Medicine. This communication simulation activity builds on didactic courses taught in Years 1–3 and serves as a summative assessment of students' behavioral health competency [22]. These courses begin in the first year and continue into the third year, providing continuous and buildable knowledge on person‐centered care, working with diverse and vulnerable populations, and preparing students to start interacting with patients [22]. Community Public Health 1, 2, and 3, which start in the first year and continue through the third year, are the cornerstones of students' learning about diverse populations and understanding their community and social responsibilities as dental professionals. A course in the second year teaches about communication skills. It includes topics related to health communications, health literacy, cultural humility, compassionate care, shared decision‐making, delivering bad news to patients, and teach‐back methods. Some of these topics are reviewed again in the behavioral health course in the third year. However, these simulation activities with diverse patients are the only time the students practice their communication and shared decision‐making in a hands‐on way. These simulation activities provide students with an opportunity to develop skills in a safe learning environment that reinforces cultural humility.

The analysis revealed notable consistency in several domains of the I‐ACT toolbox, particularly introduction, sharing information, and sustaining structure, which represent skills such as active and reflective listening, understanding others' ideas and concerns, and empathy (Table 1). These domains consistently exhibited low variability in patients and time periods, indicating that students have developed and maintained strong skills in these areas. This consistency is crucial, as effective patient introductions, clear communication of information, and the ability to build and sustain relationships are foundational to patient‐centered care [28]. Studies have shown that active and reflective listeners interpret and evaluate the patient's emotions [29, 30]. Other studies that conducted evaluations of communication skills have demonstrated that the students' performance was good at the beginning of the interview (compared to the end of the interview), where they introduced themselves and actively listened to the patient's chief complaint [31].

In contrast, the sustaining relationship and closing session domains displayed higher variability—especially the closing session, which consistently showed significant changes over time across all three patient scenarios. Importantly, this variability does not indicate regression or lack of competency. Instead, it reflects a positive trajectory in students' development of more advanced communication skills, such as summarizing key points, confirming patient understanding, and concluding interactions with empathy and professionalism. This domain assesses the student's verbal dominance and whether they prioritize language proficiency, exhibit cultural awareness, demonstrate higher emotional maturity, and respond to nonverbal cues [32, 33, 34, 35, 36].

In addition, the SPs in this study had complex health issues, such as speech comprehension issues and acute pain, which could have led to difficulty in expressing themselves. Past research has demonstrated that effective communication, particularly empathy, is essential in establishing rapport with patients experiencing acute pain [37]. In addition, Patient 2, used consistently across all 7 years, elicited the most comprehensive improvements, suggesting it is a compelling and well‐calibrated case for assessing communication.

The findings of this study have several implications for dental education, especially as communication skills training is now an essential part of dental education [38, 39]. Simulation activities support students in developing social skills, emotional intelligence, and empathy, which are instrumental in building trust with patients, understanding their needs, and providing patient‐centered care [40, 41]. Our team at the University is evaluating the I‐ACT tool and the SP methodology in light of these results to determine how we can enhance the simulation experience for students. A few suggestions would be to move the didactic portion of the curriculum closer to the simulation activity. It is taught in the second year, and the simulation activity is conducted in the third year. In addition, the importance of nonverbal cues training for students should be emphasized. In addition, the session summary is another piece of training missing from the didactic portion. We teach about teach‐back methods, but the addition of session summarization will also help students.

This study is not without limitations. The data is limited to one institution for third‐year dental students and first‐year advanced standing students; thus, the observed performance trends may not be generalizable to other dental schools. This study does not measure the student demographics and its impact on their communication skills. In addition, while using SPs provides a realistic simulation, SP encounters may not fully capture the complexities of real‐world patient interactions. A randomized controlled trial or a quasi‐experimental study (e.g., pre–post intervention study) could be performed to better understand the impact of SP simulations versus real‐world clinical rotation patient interactions on dental students' clinical performance and communication skills. In conclusion, evaluating student communication skills in a simulation setting with diverse patients over time provides valuable insights into the effectiveness of current training methods and highlights areas for improvement. By addressing the identified areas of variability, dental education programs can further enhance their students' communication skills, ultimately leading to better patient outcomes and satisfaction.
